# Supplementary material for: Distinct immunity protein families mediate compartment-specific neutralization of a bacterial toxin
Source: Cell Rep. Author manuscript; Available in PMC 2025 Dec 28. (PMC12744356; doi:10.1016/j.celrep.2025.116459)
Supplement: 1 [file NIHMS2125808-supplement-1.pdf]

Cell Reports, Volume *44*

Supplemental information

Distinct immunity protein families mediate compartment-specific neutralization of a bacterial toxin

Felicity Alcock, Yaping Yang, Justin Deme, Guillermina Casabona, Chriselle Mendonca, Fatima Ulhuq, Susan Lea, and Tracy Palmer

Document S1. Figures S1-S8, Table S1, Supplementary reference

Figure S1. Alphafold models for proteins encoded at the *esxX* locus.
A. AlphaFold multimer model for LapX3 (SAPIG0303) and LapX4 (SAPIG0304) in complex with the EsxX (SAPIG0305) LXG domain (residues 1-220). The EsxX ‘FxG’ and the LapX3 ‘FxxxD’ targeting motifs are highlighted in orange
B. AlphaFold multimer model of ExiA (SAPIG0307) with ExiB (SAPIG0308)
C. AlphaFold multimer model of ExiC (SAPIG0306) with ExiD (SAPIG0309).
Each model is shown in duplicate, coloured by subunit or by pLDDT score, and the predicted aligned error (PAE) plot is shown below each model. ‘EC’ – extracellular; ‘cyto’ – cytoplasm.

Figure S2. EsxX is unstable in the absence of LapX3 and LapX4.
S. aureus 10.1252XΔ*ess* harbouring empty pRab11, or pRab11 encoding either EsxX-Flag (pRab11-SAPIG305-flag) or LapX3-LapX4-EsxX-Flag (pRab11-303-304-305-flag) was cultured in TSB medium. When the culture reached an OD_{600} of 0.5-0.6, 500 ng/ml ATC was added to induce protein expression from pRab11. Two hours post induction whole cell samples were harvested and an equivalent number of cells (as judged by OD_{600}) were subjected to SDS PAGE, electroblotted and developed with either anti-Flag or anti-FtsZ (loading control) antibodies, as indicated.

Figure S3. Stable production of EsxX in *E. coli* requires ExiA and ExiB.
E. coli BL21(DE3) cultures carrying pACD-his305 for production of hisEsxX, together with a pTrc99-derivative plasmid producing the indicated ExiA / ExiB protein(s) were induced with IPTG. Whole cell lysates were analysed by immunoblot with antibodies against his or strep tags. EsxX was only detected in the presence of both ExiA and ExiB. *denotes a degradation product.

Figure S4. Cryo-EM analysis of EsxX complexes.
A. AlphaFold model of a LapX3-LapX4-EsxX-ExiA-ExiB pentamer coloured by subunit (i) or by pLDDT score (ii), with corresponding PAE plot.
B. Example micrograph from the LapX3-LapX4-EsxX-ExiA-ExiB complex with 2.2 µm defocus, collected as described in methods. Black scale bar, 500 Å
C. Example micrograph from the EsxX-ExiA-ExiB trimer with1.5 µm defocus collected as described in methods. Black scale bar, 500 Å
D. Representative cryo-EM 2D class averages of purified LapX3-LapX4-EsxX-ExiA-ExiB pentamer. Yellow scale bar, 100 Å.
E. Conservation analysis of 60 DUF5085 sequences identified by BLAST as homologues of the glycine zipper immunity proteins ExiB (SAPIG0308), SAR0290 or TipE (GALLO_0565).

Figure S5. *exiAB* and *exiCD* are encoded alongside *esxX* in staphylococcal genomes.
Flanking genes (FlaGs) analysis of staphylococcal *esxX* (*SAPIG0305*) homologues which are aligned and shaded black. Genes 1, 6, 3 and 2 encode proteins belonging to the DUF5079, DUF5085, DUF5085 and DUF5080 families, respectively.

Figure S6. *exiAB* co-occur with *esxX* in staphylococci
FlaGs analysis of *exiB* (*SAPIG0308*) homologues, which are aligned and shaded black. Gene 5 corresponds to *esxX.*

Figure S7. *exiCD* are found on anti-toxin islands in staphylococci
FlaGs analysis of genes coding for homologues of the DUF5079 protein ExiC (SAPIG0306), which are aligned and shaded black or orange. Genes numbered 2 and 3 correspond to additional DUF5079- and DUF5080-encoding genes respectively. Genes numbered 1 encode homologues of the nuclease immunity protein EsaG. Genes numbered 8 are *esxX* homologues, and gene pairs numbered 5-6 are *exiAB* homologues.

Figure S8. ExiAB are structurally related to GyrI.
Comparison of the ExiA-ExiB AlphaFold model and *E. coli* GyrI crystal structure (1JYH)[1]. Images on the right hand side are rotated 90° to show the binding groove between the two helices. For clarity, the ExiA structure is not shown in the rotated view.

Figure S1

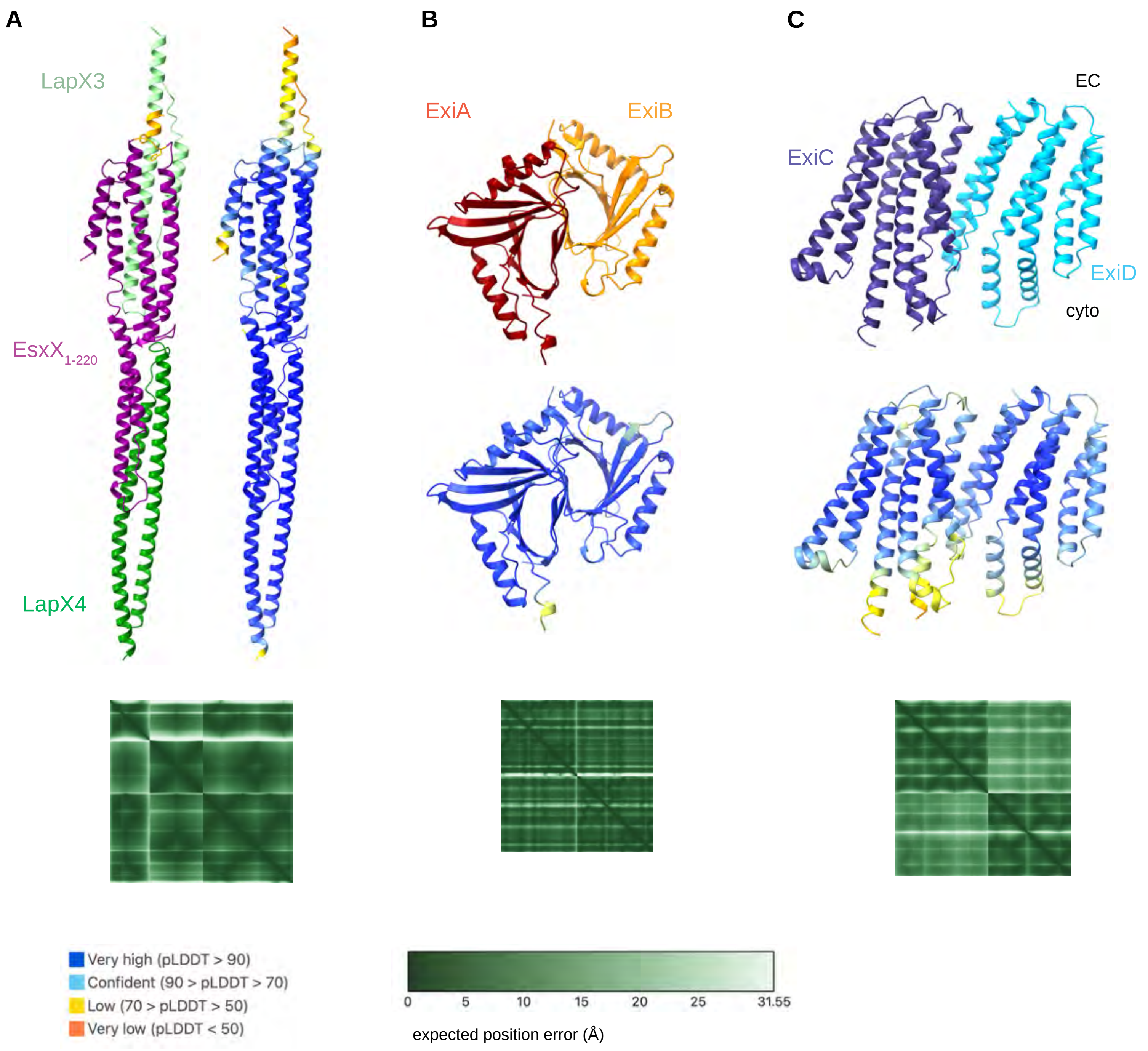
A
LapX3
EsxX1-220
LapX4
B
ExiA
ExiB
C
EC
ExiC
ExiD
cyto
Very high (pLDDT > 90)
Confident (90 > pLDDT > 70)
Low (70 > pLDDT > 50)
Very low (pLDDT < 50)
0
5
10
15
20
25
31.55
expected position error (Å)

Figure S2

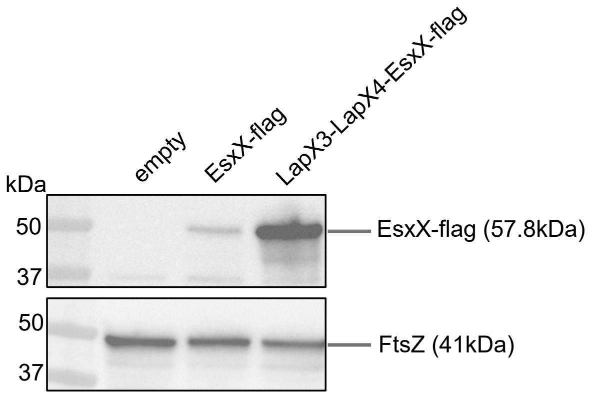
empty
EsxX-flag
LapX3-LapX4-EsxX-flag
kDa
50
37
EsxX-flag (57.8kDa)
50
37
FtsZ (41kDa)

Figure S3

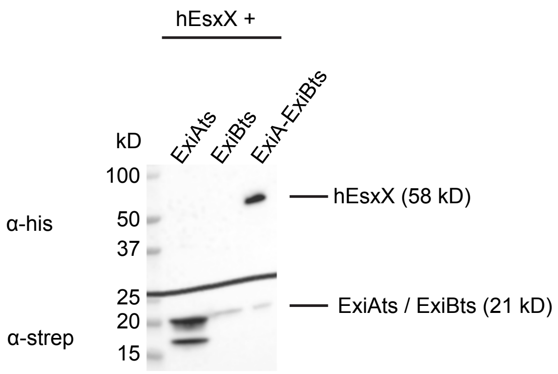
hEsxX +
ExiAts
ExiBts
ExiA-ExiBts
kD
100
50
37
25
20
15
α-his
α-strep
hEsxX (58 kD)
ExiAts / ExiBts (21 kD)

Figure S4

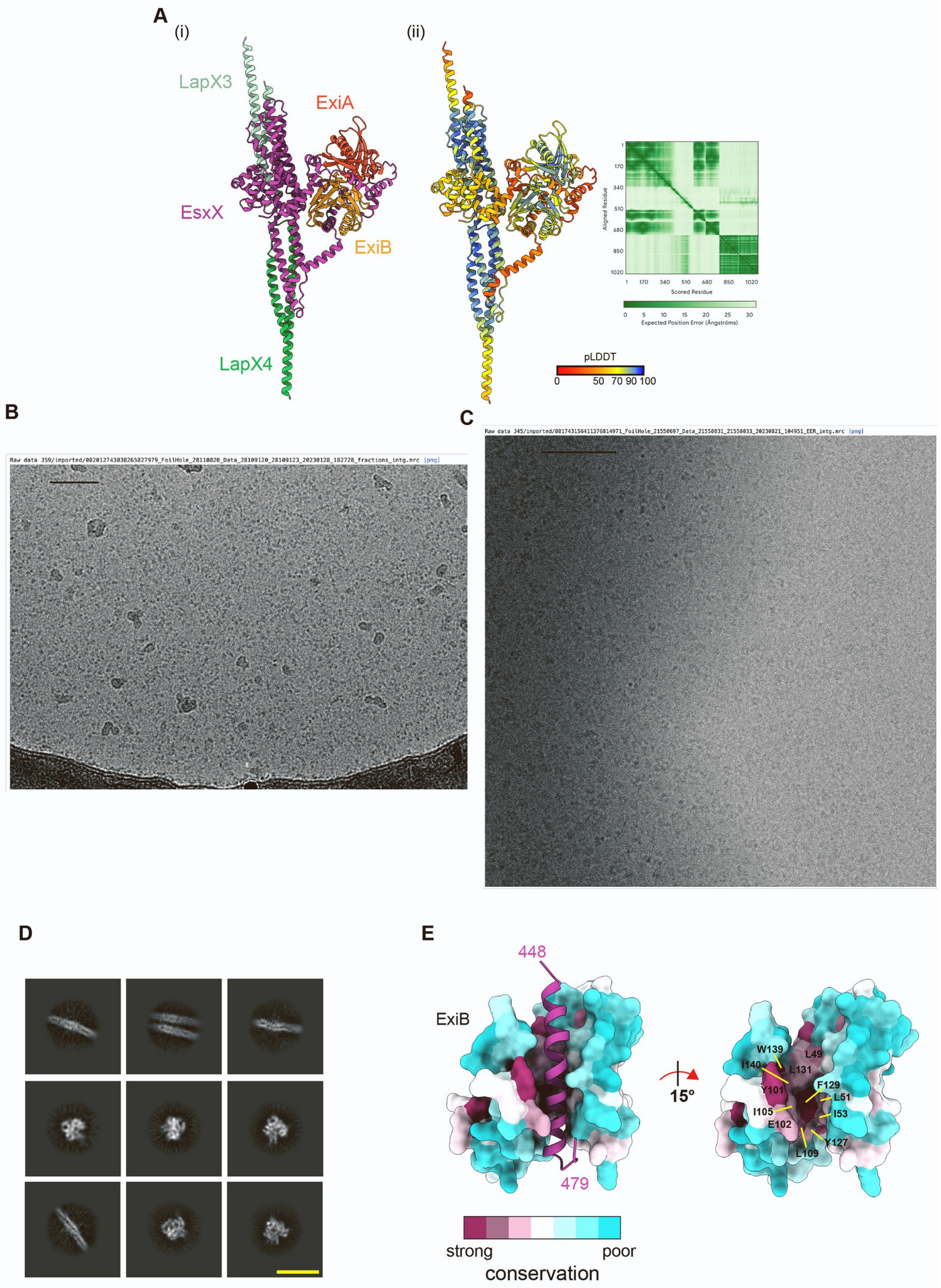
A
(i)
(ii)
LapX3
ExiA
EsxX
ExiB
LapX4
pLDDT
0
50 70 90 100
Aligned Residue
Scored Residue
Expected Position Error (Ångströms)
B
C
D
E
448
479
ExiB
15°
W139
L49
I140
L131
Y101
F129
L51
I105
I53
E102
Y127
L109
strong
poor
conservation

Figure S5

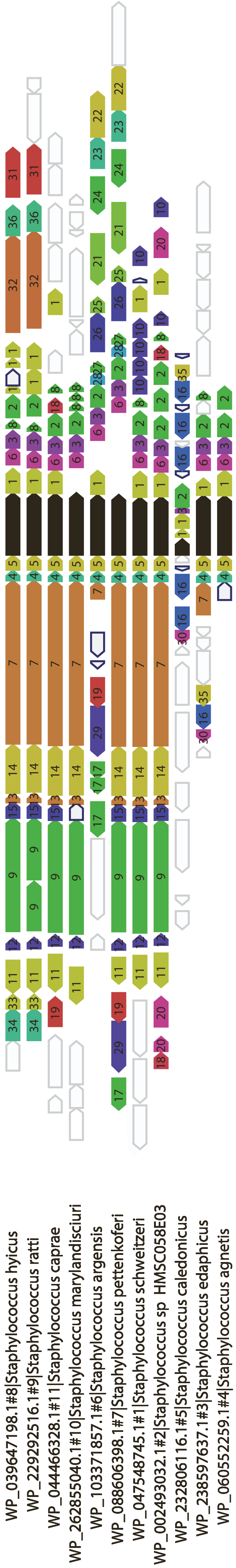

WP_039647198.1#8|Staphylococcus hyicus
WP_229292516.1#9|Staphylococcus ratti
WP_044466328.1#11|Staphylococcus caprae
WP_262855040.1#10|Staphylococcus marylandisciuri
WP_103371857.1#6|Staphylococcus argensis
WP_088606398.1#7|Staphylococcus pettenkoferi
WP_047548745.1#1|Staphylococcus schweitzeri
WP_002493032.1#2|Staphylococcus sp HMSC058E03
WP_232806116.1#5|Staphylococcus caledonicus
WP_238597637.1#3|Staphylococcus edaphicus
WP_060552259.1#4|Staphylococcus agnetis

Figure S6

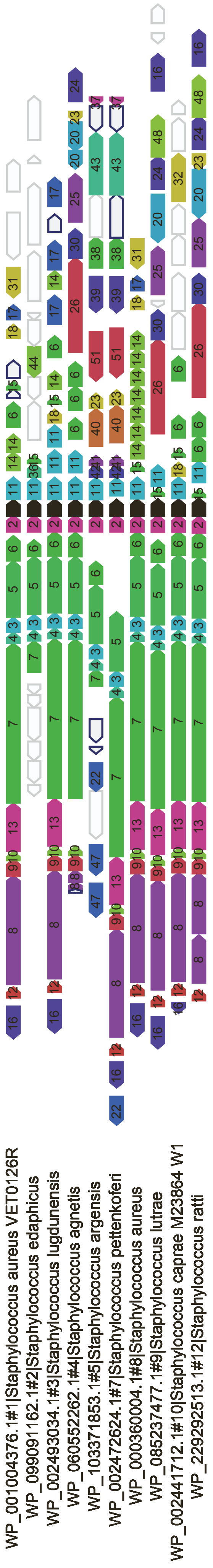
WP_001004376.1#1|Staphylococcus aureus VET0126R
WP_099091162.1#2|Staphylococcus edaphicus
WP_002493034.1#3|Staphylococcus lugdunensis
WP_060552262.1#4|Staphylococcus agnetis
WP_103371853.1#5|Staphylococcus argensis
WP_002472624.1#7|Staphylococcus pettenkoferi
WP_000360004.1#8|Staphylococcus aureus
WP_085237477.1#9|Staphylococcus lutrae
WP_002441712.1#10|Staphylococcus caprae M23864 W1
WP_229292513.1#12|Staphylococcus ratti

Figure S7

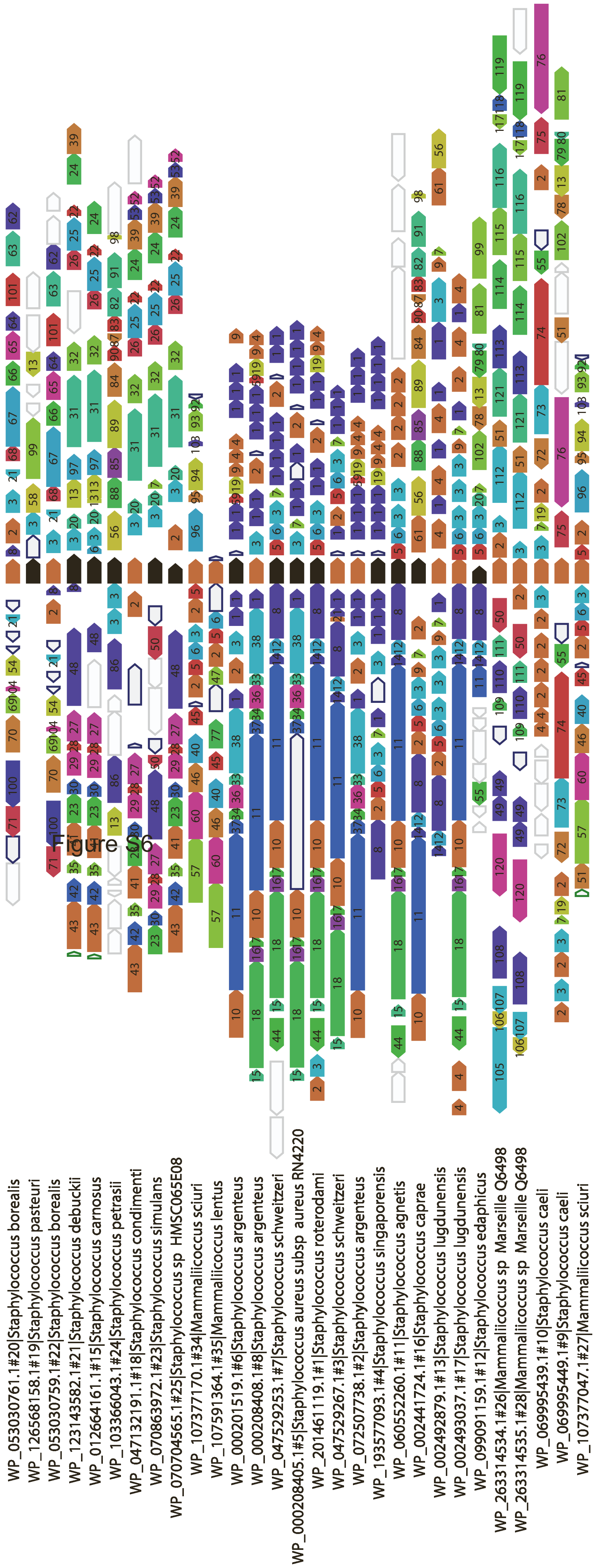
Figure S6
WP_053030761.1#20|Staphylococcus borealis
WP_126568158.1#19|Staphylococcus pasteuri
WP_053030759.1#22|Staphylococcus borealis
WP_123143582.1#21|Staphylococcus debuckii
WP_012664161.1#15|Staphylococcus carnosus
WP_103366043.1#24|Staphylococcus petrasii
WP_047132191.1#18|Staphylococcus condimenti
WP_070863972.1#23|Staphylococcus simulans
WP_070704565.1#25|Staphylococcus sp HMSC065E08
WP_107377170.1#34|Mammaliicoccus sciuri
WP_107591364.1#35|Mammaliicoccus lentus
WP_000201519.1#6|Staphylococcus argenteus
WP_000208408.1#8|Staphylococcus argenteus
WP_047529253.1#7|Staphylococcus schweitzeri
WP_000208405.1#5|Staphylococcus aureus subsp aureus RN4220
WP_201461119.1#1|Staphylococcus roterodami
WP_047529267.1#3|Staphylococcus schweitzeri
WP_072507738.1#2|Staphylococcus argenteus
WP_193577093.1#4|Staphylococcus singaporensis
WP_060552260.1#11|Staphylococcus agnetis
WP_002441724.1#16|Staphylococcus caprae
WP_002492879.1#13|Staphylococcus lugdunensis
WP_002493037.1#17|Staphylococcus lugdunensis
WP_099091159.1#12|Staphylococcus edaphicus
WP_263314534.1#26|Mammaliicoccus sp Marseille Q6498
WP_263314535.1#28|Mammaliicoccus sp Marseille Q6498
WP_069995439.1#10|Staphylococcus caeli
WP_069995449.1#9|Staphylococcus caeli
WP_107377047.1#27|Mammaliicoccus sciuri

Figure S8

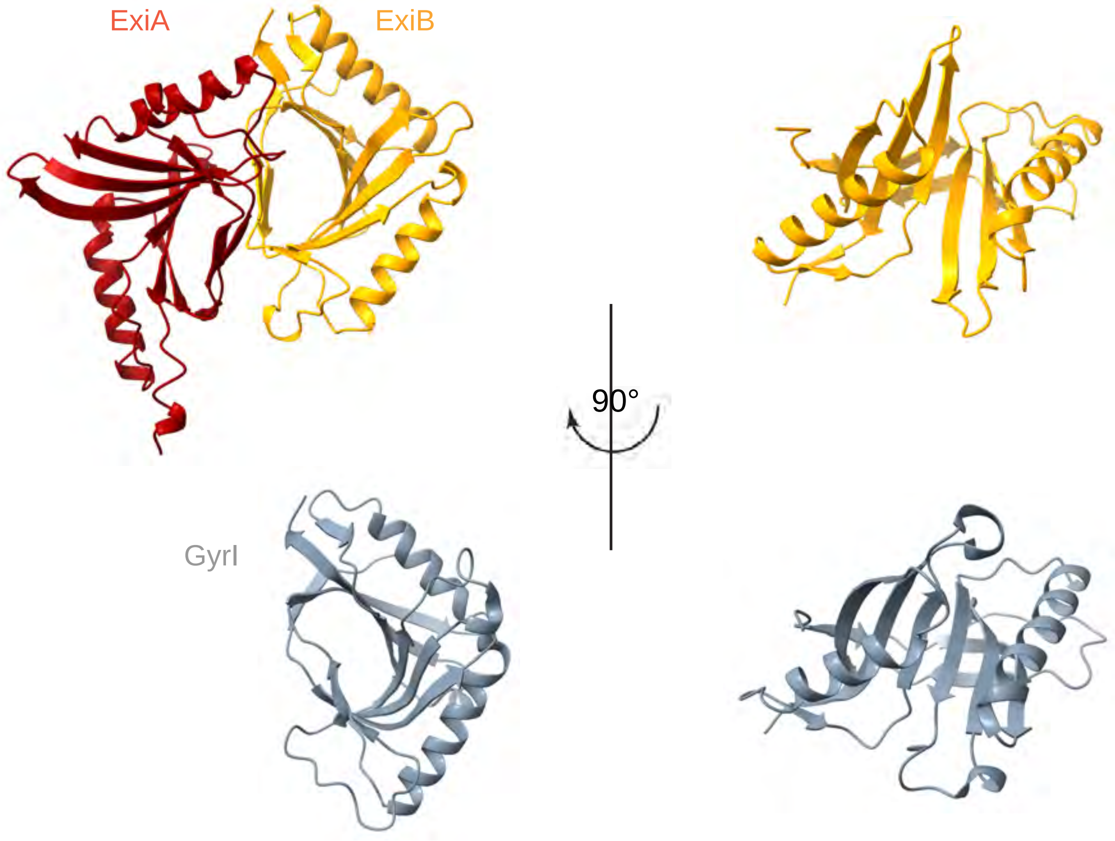

Table S1. Oligonucleotides used in this work

Name	Sequence	Template	Construct
MGC414	TATCGATAAGCTTGATATCGGTGAATCAATTGCGTCATTAATG	10.1252.X gDNA	pIMAY-ess-ST398
MGC415	TTCATCACCTTAATCATTGCCATAACTAGAAAC	10.1252.X gDNA	pIMAY-ess-ST398
MGC416	GCAATGATTAAGGTGATGAATTAAATTATTATTCAG	10.1252.X gDNA	pIMAY-ess-ST398
MGC417	TGGATCCCCCGGGCTGCAGGAACATTTTAATAGTATATACTTTTCTCAT ATTTTTTTG	10.1252.X gDNA	pIMAY-ess-ST398
2fwd305Nhe1C-term	GCGCGCTAGCCAGAGGAGGAGCCATGAAAATGAAATCCAGTGAGAAA TTAAAAGC	10.1252.X gDNA	pBAD18-SAPIG0305-CT
2rvs305_stop_SalI	GCGCGTCGACTTAAAATACATTGCTTAACGTTTT	10.1252.X gDNA	pBAD18-SAPIG0305-CT
FA221_SalI_307us_F	GGAGTCGACTTGGAGGAATAATAAATGGAAC	10.1252.X gDNA	pBAD305CT-307-308
FA245_SphI_308_R	GGAGCATGCTCAATATTCCATAACTTTTACTTTAATATC	10.1252.X gDNA	pBAD305CT-307-308
FA249_D308_Q5R	TCATTCCAATTCATCCTCATCTAAATTG	pBAD305CT-307-308	pBAD305CT-307
FA247_D308_Q5F	GCATGCAAGCTTGGCTGT	pBAD305CT-307-308	pBAD305CT-307
FA250_D307_Q5F	TGAGGATGAATTGGAATG	pBAD305CT-307-308	pBAD305CT-308
FA251_D307_Q5R	GTCGACTTAAAATACATTGC	pBAD305CT-307-308	pBAD305CT-308
MGC210	GCGCGTCGACCAGAGGAGGAGCCATGAATAATACTAAGG	10.1252.X gDNA	pBAD305CT-306-309
MGC211	GCGCGCATGCTTAGTCATCATCTGCTGTG	10.1252.X gDNA	pBAD305CT-306-309
FA_pBADssPB305CT _306_fwd	AAGCTTGGCTGTTTTGGC	pBAD305CT-306	pBAD305CT-306-309
FA_pBADssPB305CT _306_rev	GCATGCTTAGTCATCATCTG	pBAD305CT-306	pBAD305CT-306-309
FA_rbs309 plus ds (pTrc)_fwd	CAGATGATGACTAAGCATGCGGAATATTGAGGTGCGAATATGGAGTTC	10.1252.X gDNA	pBAD305CT-306-309
FA_rbs309 plus ds (pTrc)_rev	CCGCCAAAACAGCCAAGCTTTGCATGCCTGCAGGTCGA	10.1252.X gDNA	pBAD305CT-306-309
for_KpnI_SAPIG0305	GCGCGGTACCAGGAGGTTTCTAGTTATGGGGAATAAAATAAAAATGTC	10.1252.X gDNA	pRab11-SAPIG0305-flag
rev_SacI_flag_SAPIG 0305	GCGCGAGCTCTTACTTGTCGTCATCGTCTTTGTAGTCAAATACATTGCT TAACGTTTTACC	10.1252.X gDNA	pRab11-SAPIG0305-flag
FA40_BglII_307_F	CGAAAGATCTATGGAACTTGATGCATTAGTAATGC	10.1252.X gDNA	pRab11-307-308-309
FA101_EcoRI_309_R	CCAGAATTCTCAACTTCTTATATTATAATAATCTTG	10.1252.X gDNA	pRab11-307-308-309
FA218_pTrc_F	ATGGAACTTGATGCATTAGTAATG	pRab11-307-308-309	pRab11-303-304-305flag-306-307-308-309
FA219_pTrc_R	GGTCTGTTTCCTGTGTGAAATTG	pRab11-307-308-309	pRab11-303-304-305flag-306-307-308-309
FA220_303456_F	TTTCACACAGGAAACAGACCATGGGGAAATAAAAGTTG	10.1252.X gDNA	pRab11-303-304-305-306-307-308-309

FA217_303456_r trim	ACTAATGCATCAAGTTCCATTTATTATTCC	10.1252.X gDNA	pRab11-303-304-305-306-307-308-309
FA234_305flag_Q5F	GATGATGATAAATAAAAGGAGATTTAAAATGAATAATAC	pRab11-303-304-305-306-307-308-309	pRab11-303-304-305flag-306-307-308-309
FA235_305flag_Q5R	ATCTTTATAATCAAATACATTGCTTAACGTTTTAC	pRab11-303-304-305-306-307-308-309	pRab11-303-304-305flag-306-307-308-309
FA272_pRabflag_D306-9_Q5F	TATAAGAAGTTGAGAATTCTATC	pRab11-303-304-305flag-306-307-308-309	pRab11-303-304-305flag
FA273_pRabflag_D306-9_Q5R	TTTAAATCTCCTTTTATTTATCATC	pRab11-303-304-305flag-306-307-308-309	pRab11-303-304-305flag
FA301_D3078_F	TTATGGAATATTGAGGTGC	pRab11-303-304-305flag-306-307-308-309	pRab11-303-304-305flag-306-309
FA302_D3078_R	GTGTGTTTTGTAAATCTATATCATTTAG	pRab11-303-304-305flag-306-307-308-309	pRab11-303-304-305flag-306-309
FA293_p345flag_dsF	GAAGTTGAGAATTCTATCCATATG	pRab11-303-304-305flag	pRab11-303-304-305flag-307-308
FA294_p345flag_dsR	CTCCTTTTATTTATCATCATCATC	pRab11-303-304-305flag	pRab11-303-304-305flag-307-308
FA295_rbs3078_F	ATGATGATAAATAAAAGGAGGTTTTTCACTGCTTTTATATTTTAAATTG	10.1252.X gDNA	pRab11-303-304-305flag-307-308
FA296_rbs3078_R	TGGATAGAATTCTCAGCTTCTCAATATTCCATAACTTTTACTTTAATATC	10.1252.X gDNA	pRab11-303-304-305flag-307-308
pRab_fwd	AATTCACTGGCCGTCGTTTTAC	pRab11	pRab11-303-304-305flag-307
pRab_rev	GTTAACGGTACCATCATACTCTATC	pRab11	pRab11-303-304-305flag-307
D308_fwd	AGTATGATGGTACCGTTAACTATATTCAGGAGGTTTAGATCTATG	pRab11-303-304-305flag-307-308	pRab11-303-304-305flag-307
D308_rev	AAAACGACGGCCAGTGAATTTCATTCCAATTCATCCTC	pRab11-303-304-305flag-307-308	pRab11-303-304-305flag-307
pRab_fwd	AATTCACTGGCCGTCGTTTTAC	pRab11	pRab11-303-304-305flag-308
pRab_rev	GTTAACGGTACCATCATACTCTATC	pRab11	pRab11-303-304-305flag-308
D307part1_fwd	AGTATGATGGTACCGTTAACTATATTCAGGAGGTTTAGATCTATG	pRab11-303-304-305flag-307-308	pRab11-303-304-305flag-308
D307part1_rev	AATTGATTTCTTATTTATCATCATCATCTTTATAATCAAATAC	pRab11-303-304-305flag-307-308	pRab11-303-304-305flag-308

D307part2_fwd	ATGATAAATAAGAAATCAATTTAGATGAGGATG	pRab11-303-304-305flag-307-308	pRab11-303-304-305flag-308
D307part2_rev	AAAACGACGGCCAGTGAATTTCAATATTCCATAACTTTTACTTTAATATC	pRab11-303-304-305flag-307-308	pRab11-303-304-305flag-308
CM367	CTCACTGAATTTCATTTTGGCATTAGCGACAGG	10.1252.X gDNA	pRab11-Hlass-SAPIG0305CT
CM368	CCTGTCGCTAATGCCAAAATGAAATTCAGTGAG	10.1252.X gDNA	pRab11-Hlass-SAPIG0305CT
CM369	GCGCGAGCTCTTAAAATACATTGCTTAACGTTTT	10.1252.X gDNA	pRab11-Hlass-SAPIG0305CT
CM286	GCGCGGTACCAGGAGGTTTCTAGTTATGAAAACACGTATAGTCAGC	10.1252.X gDNA	pRab11-Hlass-SAPIG0305CT
FA54_NcoI_307_F	GCTGACCATGGAACTTGATGCATTAGTAATGCC	10.1252.X gDNA	pTrc-307h
FA55_HindIII_His6307_R	TAAAGCTTTTAATGATGGTGATGATGGTGTTCCAATTCATCCTCATCTAAATTG	10.1252.X gDNA	pTrc-307h
FA54_NcoI_307_F	GCTGACCATGGAACTTGATGCATTAGTAATGCC	10.1252.X gDNA	pTrc-307-308h
FA57_HindIII_His6308_R	TAAAGCTTTTAATGATGGTGATGATGGTGATATTCCATAACTTTTACTTTAATATCG	10.1252.X gDNA	pTrc-307-308h
FA108_Q5 308ts F	TTCAGGTGGTTCATCAGCTTGGTCACACCCACAATTCGAAAAATGAGGTACCCACGTGTCG	pTrc-307-308h	pTrc-307-308ts
FA109_Q5 308ts R	CCACCACCTGAACCACCACCTTTTTCGAATTGTGGGTGTGACCAATATTCCATAACTTTTACTTTAATATCGATCC	pTrc-307-308h	pTrc-307-308ts
FA220_303456_F	TTTCACACAGGAAACAGACCATGGGGGAAATAAAAGTTG	10.1252.X gDNA	pTrc-303-304-305-306-307-308ts
FA216_303456_r	ACTAATGCATCAAGTTCCATTTATTATTCCTCCAATTTAAAATATAAAAG	10.1252.X gDNA	pTrc-303-304-305-306-307-308ts
FA218_pTrc_F	ATGGAACTTGATGCATTAGTAATG	pTrc-307-308ts	pTrc-303-304-305-306-307-308ts
FA219_pTrc_R	GGTCTGTTTCCTGTGTGAAATTG	pTrc-307-308ts	pTrc-303-304-305-306-307-308ts
FA236_his305_Q5F	CACCACCACGGGAATAAAATAAAAATGTCAGAAG	pTrc-303-304-305-306-307-308ts	pTrc-303-304-h305-306-307ts
FA237_his305_Q5R	ATGATGATGCATTTTATACTCCTTTACTCTTTTATATTTATAATTG	pTrc-303-304-305-306-307-308ts	pTrc-303-304-h305-306-307ts
FA209_BamHI_305_F	GCCAGGATCCAGGGAATAAAATAAAAATGTCAGAAGTG	10.1252.X gDNA	pACD-his305
FA210_SalI_305_R	GCTTGTCGACTTAAAATACATTGCTTAACGTTTTACC	10.1252.X gDNA	pACD-his305

Reference

1. Romanowski, M.J., Gibney, S.A., and Burley, S.K. (2002). Crystal structure of the *Escherichia coli* SbmC protein that protects cells from the DNA replication inhibitor microcin B17. Proteins *47*, 403–407.
